# A dynamic nomogram to predict invasive fungal super-infection during healthcare-associated bacterial infection in intensive care unit patients: an ambispective cohort study in China

**DOI:** 10.3389/fcimb.2024.1281759

**Published:** 2024-02-26

**Authors:** Peng Li, Yan Li, Youjian Zhang, Shichao Zhu, Yongju Pei, Qi Zhang, Junping Liu, Junzhe Bao, Mingjie Sun

**Affiliations:** ^1^ Department of Infection Control, Henan Provincial People’s Hospital, People’s Hospital of Zhengzhou University, Zhengzhou, China; ^2^ Central Intensive Care Unit, Henan Provincial People’s Hospital, People’s Hospital of Zhengzhou University, Zhengzhou, China; ^3^ Respiratory Intensive Care Unit, Henan Provincial People’s Hospital, People’s Hospital of Zhengzhou University, Zhengzhou, China; ^4^ Department of Clinical Microbiology, Henan Provincial People’s Hospital, People’s Hospital of Zhengzhou University, Zhengzhou, China; ^5^ Department of Infectious Disease, Henan Provincial People’s Hospital, People’s Hospital of Zhengzhou University, Zhengzhou, China; ^6^ College of Public Health, Zhengzhou University, Zhengzhou, China

**Keywords:** COVID-19, healthcare-associated infection, intensive care unit, invasive fungal super-infection, nomogram

## Abstract

**Objectives:**

Invasive fungal super-infection (IFSI) is an added diagnostic and therapeutic dilemma. We aimed to develop and assess a nomogram of IFSI in patients with healthcare-associated bacterial infection (HABI).

**Methods:**

An ambispective cohort study was conducted in ICU patients with HABI from a tertiary hospital of China. Predictors of IFSI were selected by both the least absolute shrinkage and selection operator (LASSO) method and the two-way stepwise method. The predictive performance of two models built by logistic regression was internal-validated and compared. Then external validity was assessed and a web-based nomogram was deployed.

**Results:**

Between Jan 1, 2019 and June 30, 2023, 12,305 patients with HABI were screened in 14 ICUs, of whom 372 (3.0%) developed IFSI. Among the fungal strains causing IFSI, the most common was C.albicans (34.7%) with a decreasing proportion, followed by C.tropicalis (30.9%), A.fumigatus (13.9%) and C.glabrata (10.1%) with increasing proportions year by year. Compared with LASSO-model that included five predictors (combination of priority antimicrobials, immunosuppressant, MDRO, aCCI and S.aureus), the discriminability of stepwise-model was improved by 6.8% after adding two more predictors of COVID-19 and microbiological test before antibiotics use (P<0.01).And the stepwise-model showed similar discriminability in the derivation (the area under curve, AUC=0.87) and external validation cohorts (AUC=0.84, P=0.46). No significant gaps existed between the proportion of actual diagnosed IFSI and the frequency of IFSI predicted by both two models in derivation cohort and by stepwise-model in external validation cohort (P=0.16, 0.30 and 0.35, respectively).

**Conclusion:**

The incidence of IFSI in ICU patients with HABI appeared to be a temporal rising, and our externally validated nomogram will facilitate the development of targeted and timely prevention and control measures based on specific risks of IFSI.

## 1 Introduction

Healthcare-associated infection (HAI) is a serious public health concern worldwide, about four-fifths of which are caused by bacteria and one-tenth by fungi (Suleyman and Alangaden, 2021). To add insult to injury, these two microorganisms are often isolated simultaneously or sequentially from critically ill patients, and their interactions in polymicrobial infections are thought to enhance virulence, which leads to worsening prognosis and increased burden (MacAlpine et al., 2023). Among them, the invasive fungal super-infection (IFSI) is thus one of the main causes of disability and death in patients with healthcare-associated bacterial infection (HABI), while its incidence has shown an increasing trend over the recent decade (World Health Organization, 2022). Despite posing a major implication for human health, IFSI has not received enough attention in terms of prevention and diagnosis. On the one hand, clinical diagnosis of HAI tends to only consider the predominant infecting bacteria, and may ignore the presence of fungi in subsequent tests with atypical symptoms, thus missing the opportunities for precise anti-infection. On the other hand, the traditional fungal culture and identification often lags behind the clinical needs, and the current non-culture rapid diagnostic methods such as colloidal gold immunochromatography, latex agglutination test and PCR detection have certain limitations in clinical use due to the differences in sensitivity and specificity (World Health Organization, 2022). In addition, the lack of quick and accurate identification of IFSI also contributes to the rapid emergence and rise of antifungal resistance (Fisher et al., 2022). Although the consensus definition of invasive fungal infection (IFI) formulated and updated by the European Organization for Research and Treatment of Cancer and the Mycoses Study Group Education and Research Consortium (EORTC/MSGERC) is originally intended to be used for antifungal drug evaluation and diagnostic testing and is not recommended for clinical practice (Donnelly et al., 2020), a significant number of medical organizations and hospitals still recommend the prophylactic and therapeutic use of antifungal agents in their own IFI treatment strategies for patients with “probable” and “possible” IFI as defined in the consensus. This uncertain probability of IFI in the “probable” and “possible” categories developing to the “proven” category would potentially lead to antifungal drug abuse and increase the burden of resistance. In view of the above, how to identify and predict the occurrence of IFSI early and adequately is a major clinical challenge. Up to now, there have been a number of studies on the pathogenesis and risk factors of primary IFI in different populations and with different underlying diseases, while no researchers have, to our knowledge, reported the predisposing factors and predicted the probability of IFSI that occurred during hospitalization.

We analyzed the epidemiological characteristics of IFSI occurring in intensive care unit (ICU) patients with HABI, explored the propensity to infection by developing a simple but effective nomogram, and aimed to facilitate targeted clinical decision making as well as timely interventions implementing based on specific risk scores of IFSI.

## 2 Materials and methods

### 2.1 Study design and data collection

This observational ambispective cohort study was conducted at Henan Provincial People’s Hospital, a 5,100-bed (of which 375 are in 14 ICUs) university-affiliated institution that provides broad and specialized surgical, medical and intensive care for a population of 150 million in central China. We screened patients older than 18 years who admitted to ICU (≥ 48 hours) between Jan 1, 2019 and June 30, 2023, with the exception of Dec 7, 2022 to Jan 11, 2023, the period from the adjustment of COVID-19 prevention and control policy to the decline of the epidemic peak in Henan Province. Eligible patients were those who had been diagnosed proven with HABI by confirmatory bacterial cultures that have responsible pathogens identified. Otherwise, patients who are considered possible bacterial infection based solely on clinical symptoms, imaging findings or response to antimicrobial therapy were not considered sufficient for this study.

44 parameters were collected prior to IFSI (**Supplementary Table 1**), of which clinical features were obtained from Hospital Information System (HIS), microbiological and drug susceptibility test results were extracted from Laboratory Information System, and epidemiological characteristics of HAI were collected from Nosocomial Infection Surveillance System (NISS).

The Ethics Committee of Henan Provincial People’s Hospital approved this project (Registration number: HNSRY2023-37). And as a result of its nature as a non-interventional observational study, the Committee waived the requirement to obtain the informed consent for individual patients.

### 2.2 Procedures

We divided the entire dataset into derivation and validation cohorts in chronological order. Data collected retrospectively from Jan 1, 2019 to Dec 6, 2022, served as a model derivation cohort for model fitting and internal validation. The data prospectively collected from Jan 12 to June 30, 2023, was then used as a model validation cohort for the temporal external validation of the models built in the previous derivation cohort.

The bacterial specimens were cultured using Blood, Chocolate or McConkey agar plates at 37°C and 5% CO_2_ after gram staining. And the fungal specimens were cultured using Sabouraud dextrose agar plate after fluorescent staining, while villous colonies were transferred into Potato dextrose agar plate to observe the morphology. Then MALDI-TOF-MS (Bruker, Germany) was used for strain identification. Phenix M50 (BD, USA) and ATB FUNGUS 3 (BioMérieux, France) were used for antibacterial and antifungal agents susceptibility test, respectively. Escherichia coli strain ATCC 25922, Pseudomonas aeruginosa strain ATCC 27853, Streptococcus pneumoniae strain ATCC 49619, Staphylococcus aureus strain ATCC 29213 and Candida albicans strain ATCC 10231 were used as controls. Minimum inhibitory concentrations were determined according to the standards developed by the Clinical Laboratory Standards Institute.

### 2.3 Outcome and exposure definitions

HAI was diagnosed based on Nosocomial Infection Diagnosis Standard (NIDS) published by National Health Commission of China. And IFI was diagnosed based on the Criteria for Proven Invasive Fungal Disease published by EORTC/MSGERC, where the diagnoses for patients admitted in 2020 and before refer to the 2008 version (Pauw et al., 2008), and the diagnoses for patients admitted in 2021 and after refer to the 2020 updated version (Donnelly et al., 2020). Then if the diagnosis of IFI was at the time of or within 48 hours after HABI diagnosis, these infections were defined as parallel infections. If the diagnosis of IFI occurred ≥ 48 hours after HABI diagnosis, these infections were defined as secondary infections. Both parallel and secondary infections were classified as IFSI during HABI.

Furthermore, some exposure factors were defined as follows: (1) Multidrug resistant organisms (MDRO) were defined in accordance with the Consensus Statement published by the European Centre for Disease Prevention and Control (CDC) and US CDC (Martin-Loeches et al., 2015). (2) Combination of priority antimicrobials was defined as the use of two or more antimicrobials in the priority list published by National Institute of Hospital Administration of China, which includes carbapenems (imipenem, meropenem, panipenem, biapenem and ertapenem), glycopeptides (vancomycin and teicoplanin), tigecycline, linezolid, polymyxin, and cefoperazone sulbactam. (3) Unreasonable prescription of antimicrobials was defined as non-compliance with at least one of the following rules: (i) documented antibacterial indication, (ii) appropriate collection of samples for microbiological test, (iii) reasonable dose of antimicrobial, (iv) reasonable course of antimicrobial therapy, (v) appropriate de-escalation strategy (Timsit et al., 2020). The Antimicrobial Management Committee of the hospital where this study was conducted assessed the unreasonable prescription of antimicrobials through the electronic prescription records very two weeks under above five rules. (4) Delayed HABI reporting: For patients who meet the NIDS mentioned above, NISS will automatically send a warning message to the corresponding doctor through HIS, who will then clinically confirm or rule out the infection. If the interval between systematic warning and clinical diagnosis was more than 24 hours, the HABI report was defined as delayed. (5) During admission to HABI: The interval between the time of admission to ICU and the time when a positive specimen of the HABI-causing bacteria was sent for culturing.

### 2.4 Statistical analysis

The sample size was based on the data available for this cohort study. Given its small proportion in the dataset (< 1‰ for each variable), the missing values of qualitative data were filled with mode, and that of quantitative data were filled with mean or median. Then descriptive statistics were reported as proportions or frequencies for qualitative data, and mean (standard deviation, SD) or median (inter-quartile range, IQR) in cases of non-normal distribution for quantitative data. Chi-square test or Fisher’s exact test was used for categorical variables, and Student’s t test or Mann-Whitney U test was used for continuous variables in univariate analysis between IFSI and non-IFSI groups.

In the model-building process, considering that there were quite a number of potential covariates in this study and the multicollinearity existed among part of them, which was tested by calculating the variance inflation factors, the regularization method with least absolute shrinkage and selection operator (LASSO) and the two-way stepwise method with Akaike information criterion were used for predictor selection, respectively. All variables were screened in the LASSO method, while only variables with a *P* value < 0.1 in univariate analysis were selected and subsequently refined by two-way stepwise method in the multivariate model. And logistic regression was applied to build two prediction models with the corresponding predictors selected by above two methods.

Two models’ predictive performances were internal-validated and compared in three steps. First, the receiver-operating characteristic curve (ROC) was plotted, and the area under ROC (AUC) was measured to evaluate their classification performance. Statistical significance of the changes in AUC after selecting different independent predictors were determined by DeLong’s test (DeLong et al., 1988). Second, two models’ calibration was examined by the Hosmer-Lemeshow goodness-of-fit test and compared by Loess-based calibration plots using bootstrapping to get bias-corrected estimates of predicted probability of IFSI versus proportion of actual diagnosed IFSI, as previously reported (Sun et al., 2019). Third, considering that: (i) neither AUC nor the Harrell’s concordance index were sensitive enough to the improvement of models’ discrimination when the new predictor was introduced (Pencina et al., 2008), (ii) the sensitive Net Reclassification Index evaluated the partial change in model prediction only at specific or artificial cut-off values (Alba et al., 2017), thus the Integrated Discrimination Improvement (IDI) Index based on the predicted probability of each individual patient was calculated to compare the overall change in the predictive performance of the two models, and the statistical gap was calculated by Z-statistic (Pencina et al., 2008).

The external validity of the optimal model wined from the above comparison was further assessed in the validation cohort by computing the AUC and calibration plots. In order to make the classification and corresponding intervention of IFSI risk clinically intuitive and convenient in our upcoming impact study, a web-based interactive dynamic nomogram application was then deployed with shinyapps.io (Posit Software, PBC).

**Figure 1:**
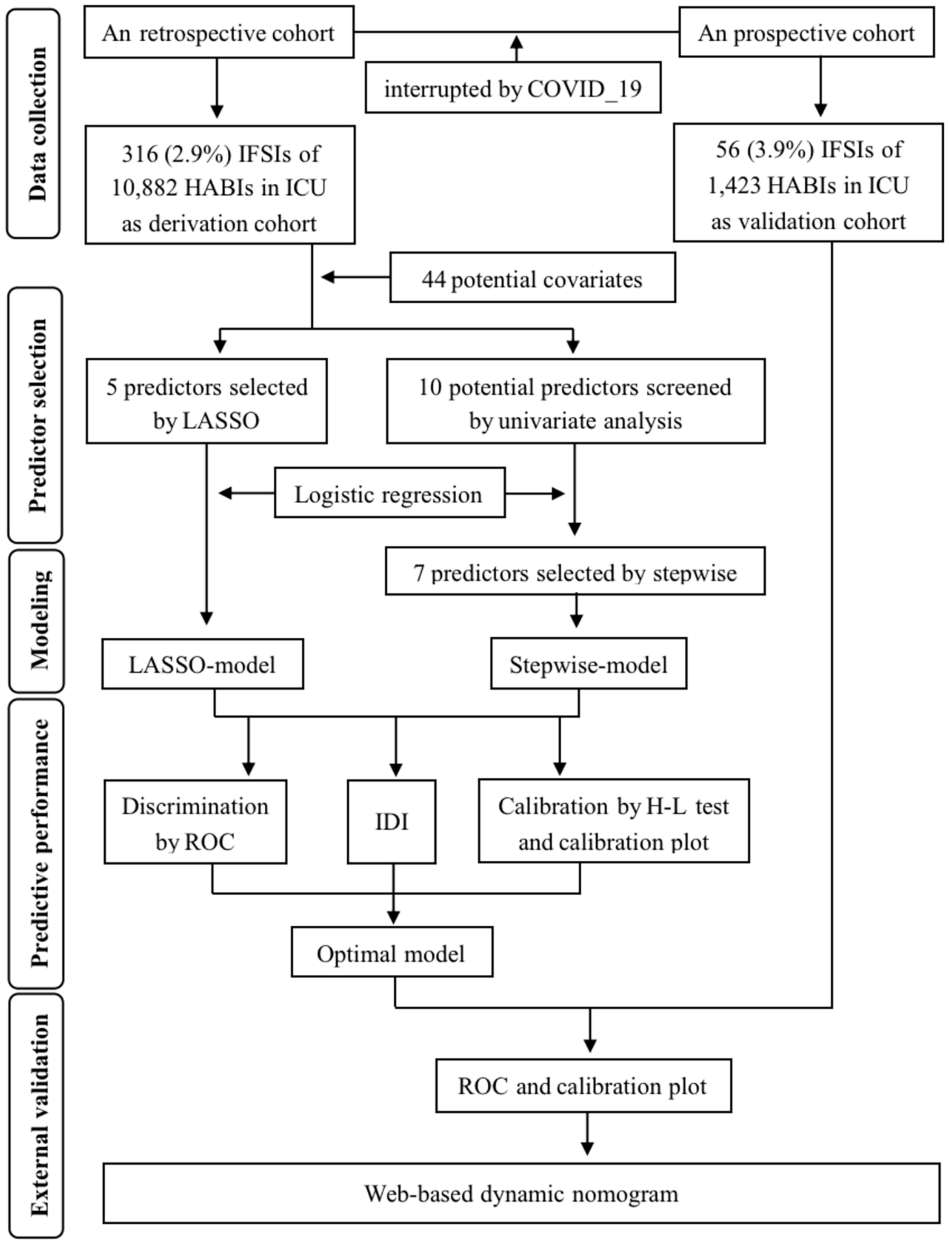
Flow diagram of prediction modeling.

The whole process of prediction modeling summarized in **Figure 1**, following the Transparent Reporting of a multivariable prediction model for Individual Prognosis Or Diagnosis (TRIPOD) statement (**Supplementary Table 2**). All statistical analyses were performed using R software (version 4.3.0), and the significance level (α) was set to 0.05 (two-tailed).

## 3 Results

### 3.1 Characteristics of infection

**Figure 2:**
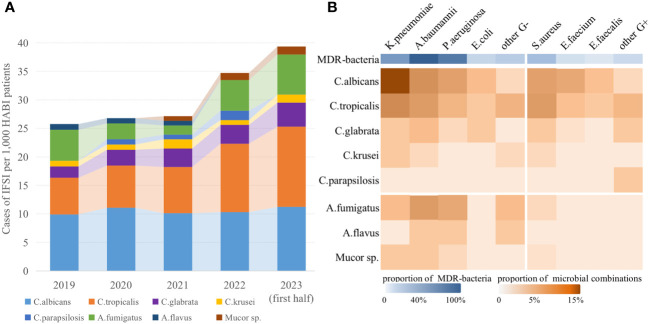
Epidemiological and microbiological characteristics of IFSI in ICU patients with HABI. **(A)** Trends in the prevalence of fungal strains causing IFSI in ICU, **(B)** Distribution of isolated pathogens among ICU patients with IFSI.

The study included a total of 12,305 patients with HABI in 14 ICUs from Jan 1, 2019 to June 30, 2023, of whom 1,423 were prospectively enrolled between Jan 12 and June 30, 2023. The main demographic and clinical characteristics of patients in deviation cohort were detailed in **Supplementary Table 1**, and the bacterial and fungal strains isolated were summarized in **Figure 2A**. Overall, 372 (3.0%) had occurred IFSI, which showed an upward trend (Mantel-Haenszel χ^2 = ^12.64, P<0.01). Among the fungal strains that caused IFSI, the most common was C.albicans (34.7%) with a decreasing proportion, followed by C.tropicalis (30.9%), A.fumigatus (13.9%) and C.glabrata (10.1%) with an increasing proportion year by year. The most combinations isolated were yeasts and gram-negative bacilli in ICU patients with IFSI (54.4%, Pearson χ^2 = ^21.98, P<0.01, **Figure 2B**). And the top five common co-isolates were C.albicans with K.pneumoniae (14.6%) and A.baumannii (6.6%), C.tropicalis with K.pneumoniae (7.2%) and S.aureus (5.4%), and A.fumigatus with A.baumannii (5.4%). There was no significant difference in the isolates of MDR-bacteria between patients with IFSI caused by yeast (59.3%) and by filamentous fungi (65.1%) (Pearson χ^2 = ^0.52, P=0.47).

### 3.2 Predictors and models

**Figure 3:**
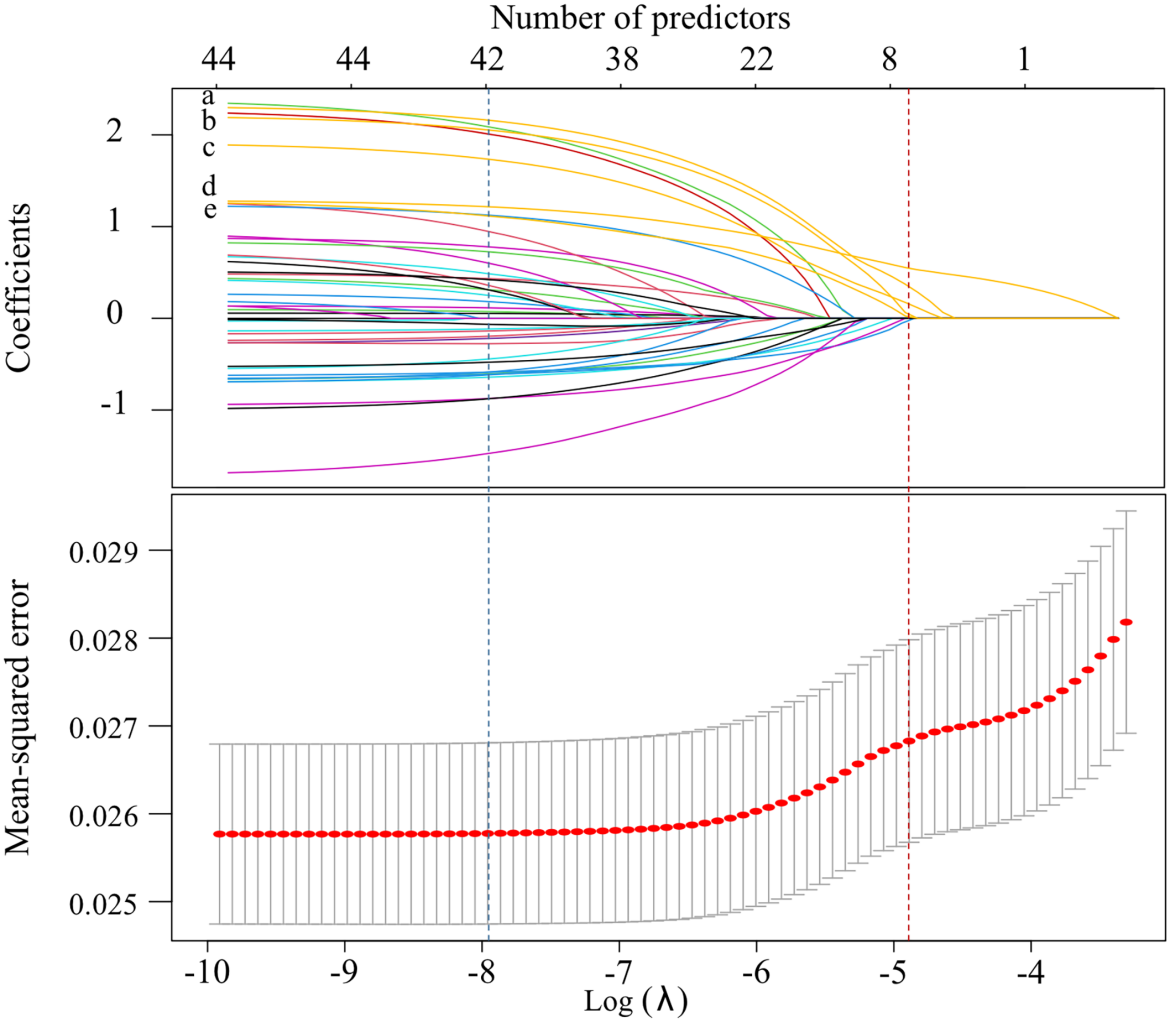
Screening process for potential predictors by LASSO method (The blue vertical dashed line showed the λ value at the minimum MSE, while the red showed the λ value at the the minimum MSE + SE. The labels from “a” to “e” represented combination of priority antimicrobials, inappropriate antimicrobials prescribing, MDRO, aCCI and S.aureus, in turn).

**Figure 4:**
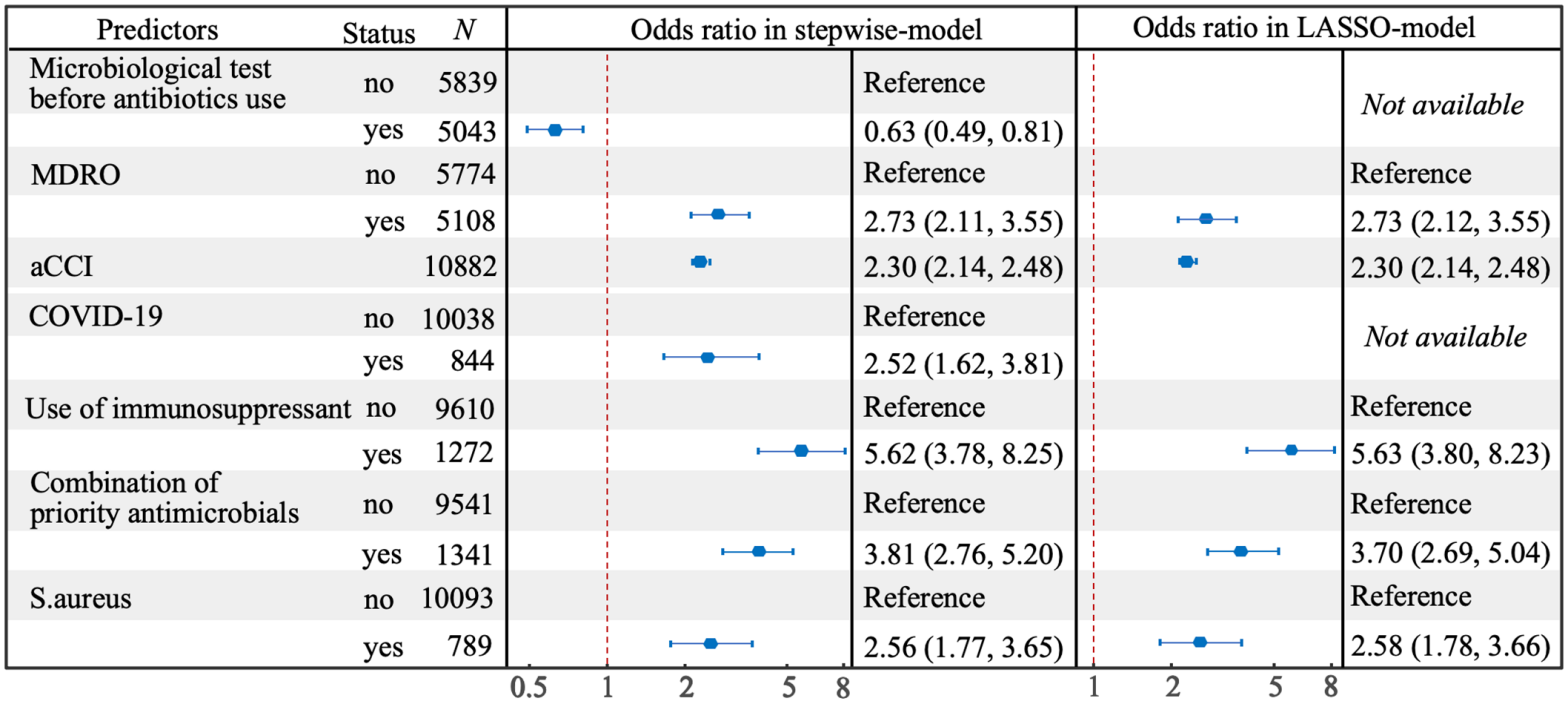
Forest plot for predictors of IFSI selected by stepwise and LASSO methods in multivariate model.

In the LASSO selection process, 41 predictors were left when the mean-squared error (MSE) was at its minimum value, while 5 predictors (combination of priority antimicrobials, use of immunosuppressant, MDRO, aCCI and S.aureus) left when the MSE was at its minimum value plus a standard error (SE), and the latter was chosen to build a more concise LASSO-model (**Figure 3**). In the stepwise selection process, 10 potential predictors were initially screened by univariate analysis (P<0.10, **Supplementary Table 1**), and 7 of which (COVID-19, microbiological test before antibiotics use and the above 5 predictors in the LASSO-model) were finally entered into the stepwise-model. The effects of predictors on IFSI were detailed in **Figure 4**.

### 3.3 Models’ performance and comparison

**Figure 5:**
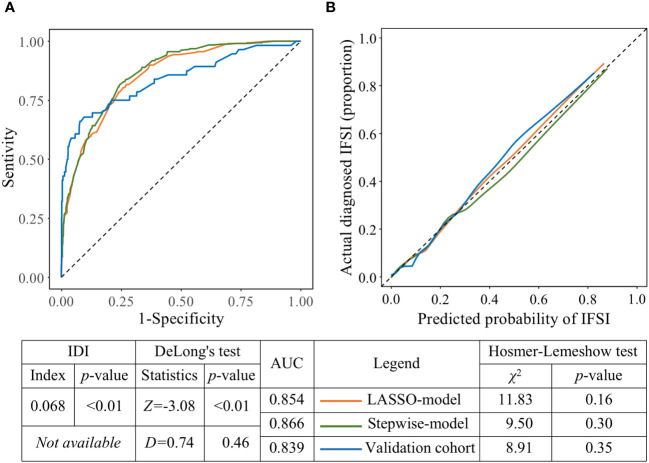
The predictive performance and external validity of models. **(A)** ROCs of the LASSO-model, stepwise-model on the derivation cohort and stepwise-model on the validation cohort, respectively. **(B)** Calibration plots of the LASSO-model, stepwise-model on the derivation cohort and stepwise-model on the validation cohort, respectively.

The LASSO-model with five predictors had an AUC of 0.854, while the stepwise-model with the addition of both COVID-19 and microbiological test before antibiotics use had a significantly higher AUC of 0.866 (P<0.01, DeLong’s test, **Figure 5A**). And the overall discriminative ability was improved by 6.8%, which indicated that stepwise-model was better than LASSO-model in classifying ICU patients with or without IFSI. The calibration plots (**Figure 5B**) showed that, for patients with a probability of IFSI greater than about 0.3, the LASSO-model predicted a slightly lower IFSI frequency than the proportion of actual diagnosed IFSI, while stepwise-model done the opposite. But these gaps were not statistically significant (P=0.16 and 0.30, respectively, Hosmer-Lemeshow test).

### 3.4 External validation and nomogram deployment

Compared with the AUC of stepwise-model in derivation cohort, the AUC of that in validation cohort decreased to 0.839 without statistical significance (P=0.46, DeLong’s test, **Figure 5A**). And there was a good fit between calibration plot and ideal curve, no significant gap existed between the frequency of IFSI predicted by stepwise-model and the proportion of actual diagnosed IFSI in validation cohort (P=0.35, Hosmer-Lemeshow test, **Figure 5B**). An interactive web-based dynamic nomogram app was then deployed, which could be accessed at “https://no79.shinyapps.io/prediction_ifsi/”, or by scanning the Quick Response code provided in **Supplementary Figure 1**.

## 4 Discussion

### 4.1 Major findings and advantages

Co-isolate of bacteria and fungi not only makes the infection develop rapidly and difficult to diagnose and treat, but also makes the risk factors complex and difficult to identify, and polymicrobes are thought to act synergistically to produce more virulent infections (Henry et al., 2017). Upon these, the global health threat of widespread anti-bacterial resistance is compounded by the under-recognized but rising fungal infection. Against the backdrop of this emerging superimposed challenge, we pioneered the epidemiological exploration of the risk and probability of IFSI in ICU patients with HABI. Our research revealed that the average incidence of IFSI was 3.0% and appeared to be a temporal rising over the past nearly five years, and A.fumigatus and yeasts other than C.albicans were playing an increasingly important role year by year in this trend. The nomogram deployed according to the optimal model demonstrated that patients with combined usage of priority antimicrobials, immunosuppressant usage, HABI caused by MDR-bacteria strains and S.aureus strains, higher aCCI, and COVID-19 had a higher risk of IFSI, while microbiological pathogenic testing before the use of antibiotics could lower the risk of IFSI.

This study has several advantages over previous studies of HAI predictors. Firstly, besides the external validation in a prospective cohort, we also improved the extrapolation of the findings through patient selection. Our study included a large number of critically ill patients over four and a half years, and ruled out the peak period of COVID-19 infection. Since most of the patients admitted to ICU during this period had COVID-19, the disease spectrum (Yang et al., 2022), clinical treatment pathways (Cai et al., 2022), and HAI prevention and control measures differed to some extent from those of conventional period (Grasselli et al., 2021), which would interfere with the temporal extrapolation of our findings (ie, the prevalence and microbiological characteristics of IFSI, the application of dynamic nomogram). Secondly, from both variable selection and model evaluation, we looked for the optimal model with concise predictors and proper predictive performance: (1) Two variable selection methods which can effectively eliminate multicollinearity and reduce dimension for a large number of variables were adopted. The LASSO procedure uses shrinkage property to results in more concise and stable predictor selection, while the stepwise procedure identifies predictors rely on statistical significance to make the model clinically meaningful (Au et al., 2020). By combining the strengths of these two methods and comparing the models they built, the key predictors of IFSI in our cohort were accurately identified. (2) In addition to the common methods for evaluating discrimination and calibration used in most predictive model studies (Alba et al., 2017), we also calculated the IDI index, which is more sensitive and comprehensive to quantitatively compare the performance of the two models. Lastly, unlike the classical nomograms developed by previous studies (Iasonos et al., 2008), which required manual calculation of the risk score of outcomes, we integrated it in an online risk calculator that presented the IFSI probability directly based on the specific predictor characteristics of patients, making it easier and more intuitive to assist in IFSI risk classification and corresponding intervention.

### 4.2 Comparison and explanations

The four dominant strains that our findings revealed to cause IFSI were all in the critical and high priority group of the WHO fungal priority pathogens list 3, and their proportion and changing trends were basically consistent with the results of the global distribution of fungi causing healthcare-associated IFI over the last 20 years shown by the SENTRY Antifungal Surveillance Program (Pfaller et al., 2019). In view of the alarming trend of IFSI and the lack of safe and effective antifungal drugs, with only a few others being under development (Hoenigl et al., 2021), more attention should be paid to its prevention while bolstering the antifungal pipeline.

In our study cohort, 39 (4.2%) of 930 COVID-19 patients with HABI developed IFSI in ICU, and no other comparable studies have been available. In spite of this, it is worth mentioning that a meta-analysis with most data from Central China in the early stage of pandemic found that only 8.1% of critically ill COVID-19 patients developed bacterial super-infections (Langford et al., 2020), while several recent studies from different countries have reported a high incidence of HAI in ICU patients with COVID-19, with an overall rate of 65.5% (range, 50.4%-76.4%) (Brandi et al., 2022; Caiazzo et al., 2022; Ćurčić et al., 2022; Hesselle et al., 2022; Novacescu et al., 2022), among which the rate of healthcare-associated IFI ranged from 5.3% to 14.6% (Brandi et al., 2022; Caiazzo et al., 2022; Novacescu et al., 2022). And our model went on to reveal that COVID-19 patients were more likely to acquire IFSI during HABI. The reason for this is unclearit is tempting to speculate that the ignorance of the antimicrobial stewardship (AMS) and some immunologic factors play a role. On the one hand, a significant number of the AMS strategies developed and implemented in the past have been conspicuously ignored as the pandemic spread (Waele et al., 2021), and in a highly cited review (Rawson et al., 2020), 72.1% of COVID-19 patients were reported to have antimicrobial therapy received, while only 7.7% of them developed a super-infection during hospitalization. On the other hand, the attack of SARS-CoV-2 and the consequent dysregulation of host’s immune response caused damage to lung epithelial cells, making the body more vulnerable to the opportunistic fungi, which may be similar to the induced super-infection mechanism of influenza virus (Verweij et al., 2020). However, it has also been hypothesized that the incidence of IFSI caused by COVID-19 may be lower than that of influenza, given that these two viruses act differently both on the receptors of host cells and on the host immune responses (Bassetti et al., 2020). In addition, the effect size of COVID-19 itself on the risk of IFSI is likely to be underestimated, as in-hospital COVID-19 isolation measures also slowed horizontal transmission of fungi between patients (Grasselli et al., 2021). All in all, these suggested that sufficient clinical attention should be paid to the prevention and control of IFSI in ICU patients with COVID-19.

Our study also showed that ICU patients with HABI caused by MDRO were more likely to develop IFSI than that caused by non-MDRO, and three epidemiological evidences may jointly explain this finding: Firstly, as a widely verified fact, the abuse of antibacterial drugs is one of the main causes of bacterial resistance (World Health Organization, 2021). Secondly, as one of the National Goals for Improving Healthcare Quality and Safety both in 2021 and 2022, improving the compliance of the microbiological pathogenic testing before the use of antibiotics could not only reduce antimicrobial abuse and empiric antimicrobial therapy (Davey et al., 2017), but also lower the risk of IFSI, as our findings validated. Lastly, as shown at the beginning of the results section, the dominant strains causing IFSI were opportunistic fungi, such as yeast and A.fumigatus, which are widespread in human body or in natural environment. Under the normal circumstances, the antagonism that exists between these fungal flora and between these fungal and other bacterial flora prevents them from thriving. However, as a recognized either cause or consequence of MDRO, the use of broad-spectrum antimicrobials, especially the combined use of priority antimicrobials, could break this antagonistic relationship and cause opportunistic IFI (Baur et al., 2017).

Another finding that needs clinical attention was that the IFSI occurred two times more often with S.aureus than with other bacteria that caused HABI in ICU patients, and 80.0% of the IFSI in patients with S.aureus-induced HABI were caused by C.albicans and C.tropicalis, implying that the pathogenesis of IFSI may depend on the source of bacterial exposure. And as for why C.albicans and C.tropicalis are more likely to cause IFSI in the presence of S.aureus, part of the reason may be that the dual-species biofilms formed through symbiotic interactions reduced their susceptibility to antimicrobials (Carolus et al., 2019). Another specific explanation is that the metabolic changes in S.aureus during super-infection induced impact interactions with host immune cells and contributed to morphogenesis and cell wall remodeling in Candida species, resulting in greater immune evasion and microbial survival (Eichelberger and Cassat, 2021).

Moreover, as one of the widely recognized risk factors for primary IFI (Suleyman and Alangaden, 2021), immunosuppressant has also been found to play an important role in the occurrence of IFSI in this study. And the induction mechanisms of immunosuppressant for these two types of infections may be basically the same, that is, suppression or disorder of immune function resulting from immunosuppressant could make the body vulnerable to the opportunistic fungi (Marcos et al., 2016). Conversely, we found that the ICU admission history (Chen et al., 2023), common invasive procedures (Liu et al., 2023) and non-single room (Abad et al., 2020), which have been identified as independent risk factors for primary HAI in previous studies, did not contribute to the development of IFSI in our cohort. These findings suggest that the differences from primary HAIs should be noted when defining the role of invasive procedures or screening strategies in HABI patients with high risk of IFSI.

### 4.3 Limits

This study does have some limitations that should be acknowledged. Firstly, due to the lack of specific symptoms, IFI parallel or secondary to existing bacterial infections such as pneumonia and bacteremia may go undiagnosed or misdiagnosed partially. The indications for targeted detection of fungi at the hospital where this study conducted mainly exist in: (1) patients with negative test results for common pathogens or who have failed to respond to empiric antimicrobial therapy, (2) patients with immunocompromised or long-term use of immunosuppressant who cannot be excluded with infection, and (3) the results of traditional bacterial culture and identification are insufficient to explain the full clinical manifestations and/or response to antimicrobial therapy. These indications of not fully covering patients with IFSI may result in some causative fungal pathogens not being confirmed microbiologically. Secondly, a growing body of research confirms that the environment is one of the main drivers of One Health, along with climate change and the use of fungicides and herbicides in agriculture also contributing to IFI (Fisher et al., 2022). Due to the limitations of the electronic case data we collected, this study could not include the living and working environmental factors of patients, such as the dietary habits and occupational characteristics engaged in animal husbandry, while exploring the pathways by which key invasive human fungal pathogens acquire antimicrobial resistance and tolerance is also a major direction of our upcoming research. Thirdly, our project was carried out in ICU of a single center, and the microbial epidemiological characteristics, clinical treatment pathways and HAI prevention and control measures may vary significantly across inpatient wards, hospital levels and geographic settings, which may have directly limited the regional extrapolation of our findings. Therewith, our model should be further tested and updated as new data becomes available. Last but not the least, this non-interventional study only validated the model’s predictive performance, and the model’s clinical performance, that is, the overall effect of the model on clinical benefit, needs to be further accessed by impact studies based on clustered randomized control trail or before and after design.

## 5 Conclusions

The incidence of IFSI in ICU patients with HABI appeared to be a temporal rising over the past nearly five years. Models to predict the occurrence of IFSI were developed, compared and externally validated. Our findings validate the combination of priority antimicrobials, immunosuppressant, MDRO, S.aureus, aCCI, COVID-19 and microbiological test before antibiotics use as quantitative predictors of IFSI. Our Web-based dynamic nomogram would assist in facilitating the development of targeted and timely prevention and control measures based on specific risks of IFSI.

## Data availability statement

The raw data supporting the conclusions of this article will be made available by the authors, without undue reservation.

## Ethics statement

The studies involving humans were approved by The Ethics Committee of Henan Provincial People’s Hospital. The studies were conducted in accordance with the local legislation and institutional requirements. Written informed consent for participation was not required from the participants or the participants’ legal guardians/next of kin in accordance with the national legislation and institutional requirements.

## Author contributions

PL: Formal Analysis, Funding acquisition, Investigation, Methodology, Software, Validation, Writing – original draft, Writing – review & editing. YL: Data curation, Investigation, Resources, Visualization, Writing – review & editing. YZ: Conceptualization, Data curation, Investigation, Writing – review & editing. SZ: Investigation, Resources, Validation, Visualization, Writing – review & editing. YP: Conceptualization, Data curation, Investigation, Writing – review & editing. QZ: Conceptualization, Methodology, Software, Writing – review & editing. JL: Conceptualization, Investigation, Resources, Writing – review & editing. JB: Formal Analysis, Methodology, Software, Writing – review & editing. MS: Conceptualization, Funding acquisition, Project administration, Resources, Supervision, Writing – original draft, Writing – review & editing.

## Supplementary material

Supplementary Figure 1 The Quick Response code of IFSI prediction.
