# Supplementary material for: A dynamic nomogram to predict invasive fungal super-infection during healthcare-associated bacterial infection in intensive care unit patients: an ambispective cohort study in China
Source: Front Cell Infect Microbiol. 2024 Feb 26;14:1281759. doi: 10.3389/fcimb.2024.1281759 (PMC10925706; doi:10.3389/fcimb.2024.1281759)
Supplement: Supplementary file 1 [file Table_1.docx]

**Supplementary Table 1** Demographic and clinical characteristics of ICU patients with HABI.

| Characteristics | Derivation cohort | | |  | Validation cohort | | |
| --- | --- | --- | --- | --- | --- | --- | --- |
|  | No IFSI  (*N*= 10566) | IFSI  (*N*= 316) | *p*-value |  | No IFSI  (*N*= 1367) | IFSI  (*N*= 56) | *p*-value |
| Age (years), mean±SD * | 58 ± 16 | 65 ± 14 | <0.01 |  | 60 ± 16 | 65 ± 14 | 0.03 |
| Sex (male), *n*(%) | 6017 (56.9) | 167 (52.8) | 0.15 |  | 772(56.5) | 30(53.6) | 0.67 |
| Nation (Han), *n*(%) | 9922 (93.9) | 294 (93.0) | 0.53 |  | 1262(92.3) | 51(91.1) | 0.73 |
| aCCI, median (IQR) * | 4 (3-6) | 6 (6-8) | <0.01 |  | 4 (3-6) | 7 (6-9) | <0.01 |
| Season, *n*(%) |  |  |  |  |  |  |  |
| Spring | 2960 (28.0) | 89 (28.2) | 0.95 |  | 776(56.8) | 26(46.4) | 0.13 |
| Summer | 2845 (26.9) | 91 (28.8) | 0.46 |  | 591(43.2) | 30(53.6) | 0.13 |
| Autumn | 2507 (23.7) | 67 (21.2) | 0.30 |  | —— | | |
| Winter | 2254 (21.3) | 69 (21.8) | 0.83 |  | —— | | |
| Underlying disease, *n*(%) |  |  |  |  |  |  |  |
| Respiratory_disease * | 4086 (38.7) | 139 (44.0) | 0.06 |  | 562(41.1) | 29(51.7) | 0.11 |
| COVID-19 * | 811 (7.7) | 33 (10.4) | 0.07 |  | 85(6.2) | 11(19.6) | <0.01 |
| Circulatory disorder | 3499 (33.1) | 106 (33.5) | 0.87 |  | 450(32.9) | 18(32.1) | 0.90 |
| Neurological disorder | 2843 (26.9) | 79 (25.0) | 0.45 |  | 330(24.1) | 12(21.4) | 0.64 |
| Cancer | 1356 (12.8) | 49 (15.5) | 0.16 |  | 179(13.0) | 8(14.3) | 0.80 |
| Hematological disorder | 1101 (10.4) | 32 (10.1) | 0.87 |  | 139(10.2) | 5(8.9) | 0.76 |
| Diabetes mellitus | 1447 (13.7) | 49 (15.5) | 0.36 |  | 218(15.9) | 8(14.3) | 0.74 |
| ICU admission in 3 months, *n*(%) | 1134 (10.7) | 31 (9.8) | 0.60 |  | 150(11.0) | 7(12.5) | 0.72 |
| Admission source, *n*(%) |  |  |  |  |  |  |  |
| Other hospitals | 2216 (21.0) | 75 (23.7) | 0.24 |  | 224(16.4) | 17(30.4) | <0.01 |
| Community | 5025 (47.6) | 138 (43.7) | 0.17 |  | 716(52.4) | 21(37.5) | 0.03 |
| Emergency | 828 (7.8) | 30 (9.5) | 0.28 |  | 96(7.0) | 4(7.1) | 0.97 |
| General wards | 2491 (23.6) | 67 (21.2) | 0.33 |  | 331(24.2) | 14(25) | 0.89 |
| Single room, *n*(%) | 4727 (44.7) | 147 (46.5) | 0.52 |  | 641(46.9) | 23(41.1) | 0.39 |
| During admission to HABI (days), median (IQR) | 9 (5-17) | 9 (4-18) | 0.90 |  | 10(6-17) | 9(4-15) | 0.16 |
| HABI site, *n*(%) |  |  |  |  |  |  |  |
| Upper respiratory tract | 29 (0.3) | 2 (0.6) | 0.23 |  | 5(0.4) | 0(0) | 1.00 |
| Lower respiratory tract | 4702 (44.5) | 147 (46.5) | 0.48 |  | 653(47.8) | 30(53.6) | 0.39 |
| Blood system | 3424 (32.4) | 112 (35.4) | 0.26 |  | 385(28.2) | 17(30.4) | 0.72 |
| Nervous system | 88 (0.8) | 0 (0) | 0.19 |  | 13(1.0) | 0 (0) | 1.00 |
| Digestive system | 169 (1.6) | 7 (2.2) | 0.39 |  | 33(2.4) | 0 (0) | 0.47 |
| Urinary system | 1482 (14.0) | 45 (14.2) | 0.91 |  | 187(13.6) | 7(12.5) | 0.80 |
| Thoracic cavity | 119 (1.1) | 6 (1.9) | 0.20 |  | 15(1.1) | 0(0) | 1.00 |
| Abdominopelvic cavity | 428 (4.1) | 17 (5.4) | 0.24 |  | 53(3.8) | 2(3.6) | 1.00 |
| Skin and soft tissue | 122 (1.2) | 5 (1.6) | 0.67 |  | 21(1.5) | 0(0) | 1.00 |
| Bones and joints | 26 (0.2) | 0 (0) | 1.00 |  | 2(0.1) | 0(0) | 1.00 |

**Supplementary Table 1** Demographic and clinical characteristics of ICU patients with HABI. (continued)

| Characteristics | Derivation cohort | | |  | Validation cohort | | |
| --- | --- | --- | --- | --- | --- | --- | --- |
|  | No IFSI  (N= 10566) | IFSI  (N= 316) | *p*-value |  | No IFSI  (N= 1367) | IFSI  (N= 56) | *p*-value |
| Invasive procedures, n(%) |  |  |  |  |  |  |  |
| Mechanical ventilation | 5424 (51.3) | 169 (53.5) | 0.45 |  | 710(51.9) | 26(46.4) | 0.42 |
| Vessel catheter | 6883 (65.1) | 197 (62.3) | 0.30 |  | 924(67.6) | 40(71.4) | 0.55 |
| Urinary catheter | 9697 (90.8) | 289 (91.5) | 0.70 |  | 1209(88.4) | 49(87.5) | 0.83 |
| Surgery | 3858 (36.5) | 110 (34.8) | 0.54 |  | 476(34.8) | 22(39.3) | 0.49 |
| ECMO | 153 (1.4) | 8 (2.5) | 0.12 |  | 20(1.5) | 3(5.4) | 0.02 |
| Pathogenic bacteria of HABI, n(%) |  |  |  |  |  |  |  |
| K.pneumoniae | 3474 (32.9) | 94 (29.7) | 0.24 |  | 455(33.3) | 17(30.4) | 0.65 |
| A.baumannii | 2644 (25.0) | 77 (24.4) | 0.79 |  | 355(26.0) | 15(26.8) | 0.89 |
| P.aeruginosa | 1517 (14.4) | 48 (15.2) | 0.68 |  | 185(13.5) | 7(12.5) | 0.82 |
| E.coli ^*^ | 758 (7.2) | 14 (4.4) | 0.06 |  | 76(5.6) | 2(3.6) | 0.73 |
| B.cepacia | 388 (3.7) | 12 (3.8) | 0.91 |  | 65(4.8) | 2(3.6) | 0.93 |
| E.cloacae | 176 (1.7) | 4 (1.3) | 0.58 |  | 17(1.2) | 0(0) | 1.00 |
| S.marcescens | 109 (1.0) | 4 (1.3) | 0.69 |  | 19(1.4) | 0(0) | 1.00 |
| P.mirabilis | 65 (0.6) | 4 (1.3) | 0.28 |  | 5(0.4) | 2(3.6) | 0.03 |
| S.aureus ^*^ | 744 (7.0) | 45 (14.2) | <0.01 |  | 132(9.7) | 17(30.4) | <0.01 |
| S.epidermidis | 274 (2.6) | 4 (1.3) | 0.14 |  | 28(2.0) | 1(1.8) | 1.00 |
| S.hominis | 194 (1.8) | 8 (2.5) | 0.37 |  | 27(2.0) | 0(0) | 0.57 |
| S.pneumoniae | 189 (1.8) | 4 (1.3) | 0.49 |  | 21(1.5) | 4(7.1) | 0.02 |
| E.faecalis | 189 (1.8) | 9 (2.8) | 0.17 |  | 19(1.4) | 1(1.8) | 0.55 |
| S.haemolyticus | 77 (0.7) | 2 (0.6) | 1.00 |  | 8(0.6) | 0(0) | 1.00 |
| More than one bacteria, n(%) | 1072 (10.1) | 26 (8.2) | 0.27 |  | 150(11.0) | 8(14.3) | 0.44 |
| MDRO, n(%) ^*^ | 4918 (46.5) | 190 (60.1) | <0.01 |  | 609(44.6) | 34(60.7) | 0.02 |
| Microbiological test before antibiotics use, n(%) ^*^ | 4934 (46.7) | 109 (34.5) | <0.01 |  | 595(43.5) | 12(21.4) | <0.01 |
| Unreasonable prescription of antimicrobials, n(%) | 3692 (34.9) | 119 (37.7) | 0.32 |  | 457(33.4) | 22(39.3) | 0.36 |
| Use of immunosuppressant, n(%) ^*^ | 1225 (11.6) | 47 (14.9) | 0.07 |  | 169(12.4) | 23(41.1) | <0.01 |
| Delayed HABI reporting, n(%) | 2775 (26.3) | 91 (28.8) | 0.31 |  | 340(24.9) | 17(30.4) | 0.35 |
| Combination of priority antimicrobials, n(%) ^*^ | 1274 (12.1) | 67 (21.2) | <0.01 |  | 211(15.4) | 16(28.6) | <0.01 |
| *Variables with a p value < 0.1 in derivation cohort were selected and subsequently refined by stepwise method in multivariate model. Abbreviations: aCCI = the age-adjusted Charlson Comorbidity Index; ECMO = extracorporeal membrane oxygenation; HABI = healthcare-associated bacterial infection; IFSI = invasive fungal super-infection; MDRO = multidrug resistant organism. | | | | | | | |
