# Supplementary figures and images for: A dynamic nomogram to predict invasive fungal super-infection during healthcare-associated bacterial infection in intensive care unit patients: an ambispective cohort study in China
Source: Front Cell Infect Microbiol. 2024 Feb 26;14:1281759. doi: 10.3389/fcimb.2024.1281759 (PMC10925706; doi:10.3389/fcimb.2024.1281759)

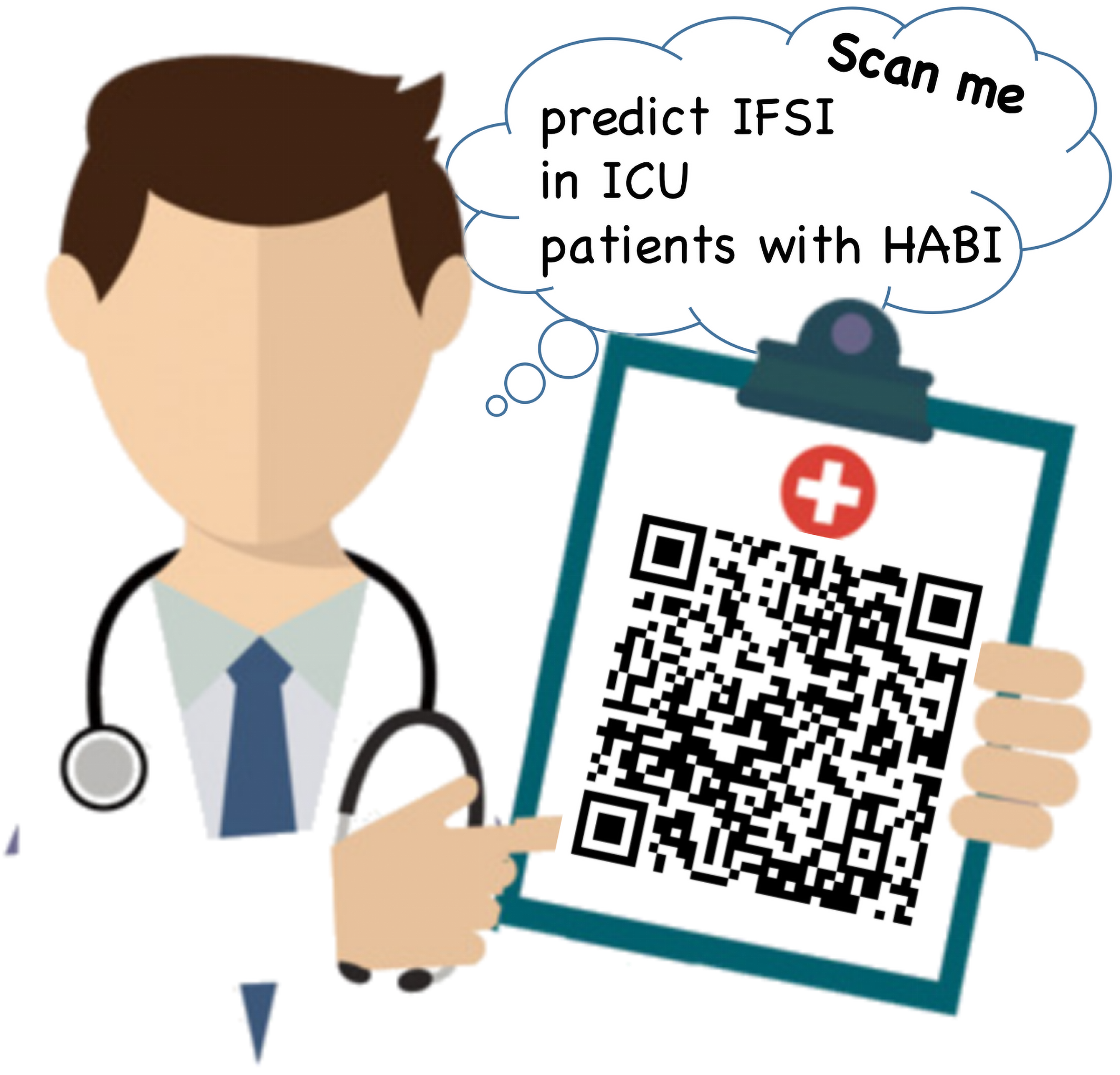

Supplement: Supplementary Figure 1 — The Quick Response code of IFSI prediction. [file Image_1.tif]
